# Binding of Equine Seminal Lactoferrin/Superoxide Dismutase (SOD-3) Complex Is Biased towards Dead Spermatozoa

**DOI:** 10.3390/ani13010052

**Published:** 2022-12-23

**Authors:** Abdorrahman S. Alghamdi, Carleigh E. Fedorka, Kirsten E. Scoggin, Alejandro Esteller-Vico, Kaylin Beatty, Gabriel Davolli, Barry A. Ball, Mats H. T. Troedsson

**Affiliations:** 1 Departments of Agriculture and Natural Resources, University of Minnesota Crookston, Crookston, MN 56716, USA; 2 Gluck Equine Research Center, University of Kentucky, Lexington, KY 40546, USA

**Keywords:** equine, spermatozoa, lactoferrin, seminal plasma, binding

## Abstract

**Simple Summary:**

Lactoferrin is found in equine seminal plasma and has been suggested to mediate binding between sperm and polymorphonuclear neutrophils (PMNs) in the female reproductive tract. The overall objective of this study was to determine if lactoferrin binds preferentially to non-viable equine sperm. Seminal plasma lactoferrin was consistently bound to superoxide dismutase (SOD-3), forming a LF/SOD-3 protein complex. Various methods of biotinylation were evaluated to optimize the method and determine binding sites of LF/SOD-3 on equine spermatozoa. The LF/SOD-3 complex bound to equine sperm with preference to non-viable spermatozoa, possibly facilitating elimination of this sperm population through PMN-phagocytosis.

**Abstract:**

Sperm-neutrophil binding is an important facet of breeding and significantly impacts fertility. While a specific seminal plasma protein has been found to reduce this binding and improve fertility (CRISP-3), additional molecule(s) appear to promote binding between defective sperm and neutrophils. Recent work has suggested one of these proteins is lactoferrin (LF), an 80 kDa iron-binding protein found throughout the body, but the purity of the protein was not confirmed. It is unknown if LF binds to sperm selectively based on viability, and if receptors for LF are located on equine sperm. To evaluate this, we attempted to purify equine seminal LF from five stallions (n = 5), biotinylate LF, and evaluate potential binding site(s) on spermatozoa. LF was consistently associated with superoxide dismutase (SOD-3), and all attempts to separate the two proteins were unsuccessful. Flow cytometric and microscopic analyses were used to compare LF/SOD-3 binding to viable and nonviable spermatozoa. Additionally, various methods of biotinylation were assessed to optimize this methodology. Biotinylation of seminal plasma protein was an effective and efficient method to study seminal plasma protein properties, and the binding site for LF/SOD-3 was found to be broadly localized to the entire sperm cell surface as well as selective towards nonviable/defective sperm. Although we were not able to determine if the binding to equine spermatozoa was through LF or SOD-3, we can conclude that equine seminal LF is tightly bound to SOD-3 and this protein complex binds selectively to nonviable spermatozoa, possibly to mark them for elimination by neutrophil phagocytosis.

## 1. Introduction

Post-breeding inflammation of the uterus is an important physiological event that involves an influx of polymorphonuclear neutrophils (PMN) into the uterine lumen [1]. The impeding neutrophils interact with sperm for effective clearance, and this is modulated by seminal plasma proteins, which appear to play an important role in PMN binding and phagocytosis of spermatozoa [2,3,4,5,6,7]. Sperm-neutrophil binding and aggregate formation are being mediated by neutrophil extracellular traps (NETs; neutrophil genomic DNA that ensnares sperm), which are degraded by seminal DNase activities that free entangled sperm, in addition to a direct cell-to-cell binding indicative of a receptor-ligand mechanism [6,8]. Doty et al. (2011) demonstrated that cysteine-rich secretory protein-3 (CRISP-3) was responsible for reducing neutrophil binding to equine sperm in a dose-dependent manner [4]. In contrast, the biological function of an additional protein was identified in seminal plasma and found to enhance sperm-neutrophil binding activity. Through protein isolation and identification, this protein was determined to be the 80 kDa iron-chelating lactoferrin (LF) [9,10,11]. However, the purity of LF was not confirmed, and preliminary data in our laboratory suggest that LF in seminal plasma is tightly bound to a 32 kDa protein identified as superoxide dismutase (SOD-3).

LF is found throughout the body in a variety of tissues and secretions, including the equine epididymis, uterus, and within neutrophil granules [12,13,14]. In investigated species, LF possesses a myriad of functions including iron metabolism/regulation, antimicrobial and antiparasitic properties, immune modulation, and enzymatic activities [12,15,16,17,18,19,20,21,22,23]. The versatile functionality of LF appears to be mediated by the various receptors which identify different domains of LF [24]. The LF receptors are proposed to be cell-specific, and this has been described in various immune cell types, including neutrophils [24]. Within the reproductive system, LF is an estrogen-dependent protein that has been implicated in sperm maturation and fertilization [25,26,27]. In the boar, LF binds to epididymal spermatozoa to assist in spermatozoal maturation [28], and in the stallion, LF was correlated with sperm concentration [29]. Additionally, endometrial expression of LF has been found to change with the stage of the estrous cycle and increase in mares with persistent breeding-induced endometritis, although this may be due to an increased presence of PMNs [13]. While the binding sites of LF on human sperm have been reported [30], this has not been examined in equines.

Superoxide dismutase exists in three isoforms (SOD-1, SOD-2, and SOD-3). All three isoforms scavenge superoxide radicals and therefore, play important roles in the protection against oxidative stress. While SOD-1 and SOD-2 are mostly found in the cytoplasm, nucleus, and mitochondria, the action of SOD-3 is mostly extracellular [31]. SOD-3 has been found in seminal plasma with some researchers observing higher levels in infertile males [23]. 

Based on previous observations, we hypothesized that LF binds to equine spermatozoa and that this binding is preferential to nonviable sperm. The objectives of this study were to (1) determine the binding sites of LF on equine sperm and (2) determine whether LF binding is preferential to live or nonviable equine spermatozoa.

## 2. Materials and Methods

### 2.1. Animal Work

All sample collections were carried out in accordance with the Institutional Animal Care and Use Committee guidelines at the University of Kentucky (2012-0981).

### 2.2. Sperm Preparation

Semen was collected from five stallions (n = 5) between 3 and 20 years of age and of varying breeds. All stallions had normal reproductive parameters and performance including sperm parameters, general health, and absence of any pathological lesions or symptoms. Semen was collected using a Missouri model artificial vagina with an in-line gel filter. All semen used had initial progressive motility of at least 70% as determined using SpermVision^®^ Computer Assisted Sperm Analysis software (Minitube USA; Verona, WI, USA). To minimize contact between seminal lactoferrin and spermatozoa, 10 mL of semen was diluted 1:4 with lactated Ringer’s solution (LRS) at 37 °C within 5 min from ejaculation and immediately subjected to centrifugation at 400× *g* for 10 min. The supernatant was decanted, and the sperm pellet was resuspended in 30 mL LRS and transported to the laboratory in an insulated container at ambient temperature (20–27 °C). The time for sperm transport from the stud barn to the laboratory ranged from 20 to 30 min. Upon arrival at the lab, the semen was centrifuged a second time and the sperm pellet was resuspended in Dulbecco’s Phosphate Buffered Saline (PBS) at a concentration of 300 × 10^6^/mL, which served as a “stock” of live sperm that was used to prepare the reactions for the different experiments. To obtain “killed” sperm (heat-treated or snap frozen), this stock was divided into two or three portions depending on the experiment. Heat-treated sperm were subjected to 50 °C for 15 min, and snap-frozen sperm were subjected to liquid nitrogen for 5 min, followed by thawing at room temperature.

### 2.3. Identification of LF in Seminal Plasma

#### 2.3.1. Isolation of Equine Seminal LF

**Figure 1:**
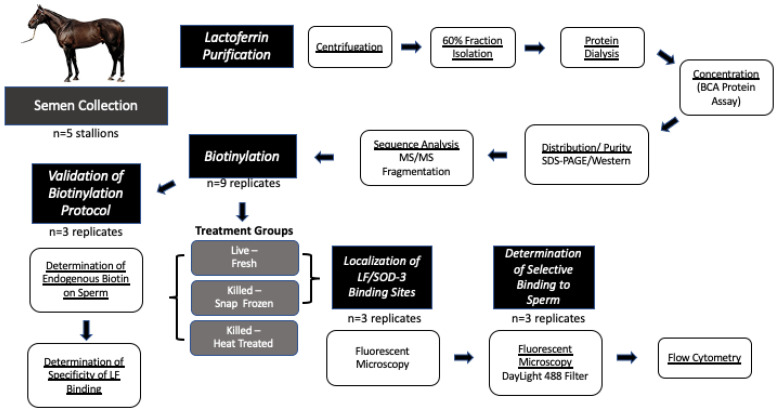
Summary of experimental design. The flowchart summarizes key steps of the experimental design.

The overall experimental design is illustrated in Figure 1. Five ejaculates were collected from each stallion as described above. Seminal plasma was separated from spermatozoa by centrifugation at 600× *g* for 15 min and the sperm pellet was discarded. The remaining seminal plasma portion was further submitted to centrifugation at (2000× *g*/15 min/4 °C) and the supernatant was pooled from all five stallions before being divided into 50 mL aliquots. Following centrifugation, phenylmethanesulfonyl fluoride (2 mL, Millipore Sigma, Burlington, MA, USA) was added to each aliquot of the pooled seminal plasma samples to inhibit any endogenous protease activity. Aliquots were stored at −20 °C until use. 

The seminal plasma samples were buffered with 25 mM sodium phosphate, pH 7.2, and protein fraction precipitated with the addition of 60% (*w*/*v*) solid ammonium sulfate at 4 °C as described in Doty et al. [4]. The 60% (*w*/*v*) ammonium sulfate fraction of seminal plasma proteins was submitted to a HiPrep Heparin Fast Flow 16/10 column previously equilibrated in 20 mM Tris-HCl (pH 7.4) containing 100 mM NaCl. After loading and washing, the bound material was eluted in a stepwise manner (4 mL fractions) with an equilibration buffer containing 0.10, 0.20, 0.30, 0.40, 0.50, and 1.0 M NaCl, respectively. Fractions were pooled based on the A_280_ elution profile and analyzed by SDS-PAGE. Lactoferrin-containing fractions were pooled and dialyzed in a dialysis cassette against 1× PBS, pH 7.2 containing 5 mM EDTA, and 0.02% (*w*/*v*) NaN_3_.

Sephacryl S-200 column (3 × 100 cm) was equilibrated and eluted with 1× PBS, pH 7.2, containing 5 mM EDTA, and 0.02% (*w*/*v*) NaN_3_. The collected fractions (6 mL) were pooled based on the A_280_ elution profile. Lactoferrin-containing fractions were pooled and dialyzed overnight at 4 °C in a dialysis cassette as described above. The samples were concentrated by Amicon Ultra Spin columns 10,000 MW cut-off (Millipore Sigma, Burlington, MA, USA). The protein concentration of the dialyzed sample was determined using the BCA Protein Assay kit (Thermo Fisher Scientific, Waltham, MA, USA). Protein distribution and purity of the isolated fractions were determined by SDS-PAGE and by Western blot using goat anti-human lactoferrin polyclonal antibody (1:1000, sc-14434, Santa Cruz Biotechnology, Inc., Santa Cruz; no longer in production) and mouse SOD-3 monoclonal antibody (1:1000, sc-58427, Santa Cruz Biotechnology, Inc., Santa Cruz, CA, USA).

#### 2.3.2. Sequence Analysis and Identification

Mass spectrometric analysis was performed at the Proteomics Core Facility of the University of Kentucky. The two bands of interest were extracted from the SDS-PAGE and digested with trypsin. The extracted peptides were desalted and analyzed by MALDI TOF/TOF. Peptide peaks of greatest intensity (which were not on the trypsin/keratin exclusion list of masses) were fragmented, and the MS/MS fragmentation data were subjected to a database search by Protein Pilot against the entire Uniprot database. All MS/MS samples were analyzed using Mascot (Matrix Science, London, UK; version 2.2.0). The 80 kDa protein band was identified as lactoferrin (30.5% coverage) and the 32 kDa protein band was identified as SOD-3 (40.2% coverage).

### 2.4. Validation of Biotinylation Protocol

#### 2.4.1. Biotinylation of Purified LF/SOD-3

Biotinylation of 2 mg of LF/SOD-3 complex was performed using a Sulfo-NHS-Biotinylation Kit (Thermo Fisher Scientific) according to the manufacturer’s instructions. Following the biotinylation reaction (2 h on ice), the excess biotin was removed using the included desalting column. The level of biotin incorporation was determined to be approximately 4.7 molecules of biotin for each molecule of LF/SOD-3 according to the recommended HABA (4′-hydroxyazobenzene-2-carboxylic acid) assay. Bovine serum albumin (BSA; 2 mg) was similarly biotinylated as a non-specific protein control with 5.8 incorporation level.

#### 2.4.2. Determination of the Presence of Endogenous Biotin on/in Equine Sperm 

To determine the presence of endogenous biotin on equine sperm, ejaculates from five stallions were collected as described above and divided into three portions. One portion was kept at room temperature (control), and the other two were subjected to heating (50 °C for 15 min) or snap-freezing (in liquid nitrogen for 5 min and thawed at room temperature) to compromise the cell membrane integrity and facilitate exposure of any endogenous biotin. Each portion was further divided into four aliquots; the first of which was not processed any further (control). The other three aliquots were incubated separately with Avidin, StreptAvidin or NeutrAvidin at 10 μg/mL (DayLight 488 conjugates, EM/EX 493/518; Thermo Fisher Scientific) at room temperature for 20 min. Wet mounts were examined with a dual visible/fluorescent microscope to determine the level of positive fluorescent staining in proportion to sperm numbers. SYBR 14 was used as a positive control for sperm fluorescence, while biotinylated-LF/SOD-3 (biot-LF/SOD-3) served as a positive validation control for the method.

### 2.5. LF/SOD-3 Binding to Equine Spermatozoa

#### 2.5.1. Determination of Specificity of LF/SOD-3 Binding

In a preliminary experiment, the non-specific binding sites on the sperm were blocked for 60 min with non-biotinylated BSA at 0, 0.1, 0.2, 0.4, 0.8, 1, 2, 3, and 5% (henceforth, non-biotinylated BSA will be referred to as BSA). The spermatozoa were then treated with biotinylated-BSA (biot-BSA; 100 μg/mL) for 50 min at room temperature. Sperm were collected by centrifugation at 400× *g*/10 min. careful and thorough removal of the supernatant, washed with 600 μL PBS by another round of centrifugation, the pellet was resuspended in 100 μL PBS containing fluorescent NeutrAvidin (10 μg/mL), and wet mounts were examined and photo-documented using a dual visible/fluorescent microscope. To ascertain the specificity of LF/SOD-3 binding to sperm (as compared to BSA binding) and to determine the appropriate blocking time, we performed a competition assay as follows: (1) No BSA or biotinylated protein was used (control for any non-specific binding of NeutrAvidin to endogenous biotin); (2) BSA and either biot-BSA or biot-LF/SOD-3 was added to the reaction at the same time (all proteins were added at 100 μg/mL); (3) BSA was added to the reaction 5, 10, 20, or 40 min before the addition of biot-BSA or biot-LF/SOD-3; and (4) No biot-BSA or biot-LF/SOD-3 was used. The reactions were incubated for 50 min after the addition of the biot-BSA or -LF/SOD-3 at room temperature followed by centrifugation at 400× *g*/10 min, washing, then resuspension in 100 μL of PBS containing fluorescent NeutrAvidin as mentioned above. These experiments were repeated three times (n = 3) with semen from three different stallion. 

#### 2.5.2. Determination of the LF/SOD-3 Binding Sites on Sperm 

Once the basic elements of endogenous biotin and on blocking conditions were determined, untreated (from the “stock” described in sperm preparation above) and killed (snap-frozen for 5 min in liquid nitrogen then thawed at room temperature) sperm were blocked with BSA for 10 min before adding biot-LF/SOD-3 (100 μg/mL) and incubating the reaction at room temperature for 50 min. The reaction was then subjected to centrifugation at 400× *g*/10 min and the sperm pellets were washed with 600 μL PBS, resuspended in 100 μL PBS containing fluorescent NeutrAvidin (6 μg/mL), and wet mounts were examined under a dual visible-fluorescent microscope. Propidium iodide (PI; EX/EM 536/617) was also added to all reactions at final concentration of 12 μM to help identify dead sperm. Photo-documentation of at least 20 randomly selected frames were captured with both visible and fluorescent microscope (using filters suitable for both PI and NeutrAvidin) and saved for later analysis and counting. Following the capture of these 20 frames, the slide (or a replacement slide if dryness occurred) were examined carefully for human-based observation with appropriate magnification and light source/filter. Each reaction was prepared to contain a final sperm concentration of 110 × 10^6^/mL in a total reaction volume of 300 μL (to facilitate visualization of sperm pellets during centrifugation), but after the incubation with biotin and the washing, sperm were resuspended in 100 mL (to conserve stains and maximize sperm density). This experiment was performed in three replicates using an equal number of sperm pooled from two stallions for each replicate (n = 3). 

### 2.6. Comparison of LF/SOD-3 Binding to Live and Killed Sperm

#### 2.6.1. Flow Cytometric Analysis 

Untreated and freeze-killed spermatozoa were treated as two separate populations, and each was divided into five aliquots in PBS containing 1% BSA (for blocking of non-specific sites) and incubated for 10 min at room temperature. One aliquot was treated with biot-LF/SOD-3, while the other four were not. After 50 min of incubation, all aliquots were subjected to centrifugation and washing as described above. After washing, the sperm that was incubated with biot-LF/SOD-3 was resuspended in PBS containing PI and NeutrAvidin, while the other four that were not treated with biot-LF/SOD-3 were resuspended in: (1) PBS alone; (2) PBS with PI; (3) PBS with NeutrAvidin; (4) PBS with PI and NeutrAvidin. PBS alone was used to determine the non-specific light scatter, which was essentially absent. Sperm in PBS alone was used to set the sperm gate around the main population determined by side (SSC) and forward (FSC) scatter density plot (step 1; gate P3). Following gating on spermatozoa based on SSC-FSC, a density plot with FL1 (Daylight 488) and FL3 (PI) and both were determined for background fluorescence in quadrants (Figure S1). For each treatment, at least 20,000 events were acquired, and this experiment was repeated three times (n = 3) each of which was performed with sperm pooled from two different stallions. 

#### 2.6.2. Microscopic Analysis 

Live and freeze-killed sperm were treated separately according to the method described above. LF/SOD-3 positive (LF+) sperm stain green while PI positive (PI+) sperm stain red. Since the 20 frames from each replication were captured by visible overlay with DayLight 488 filter (to capture whole sperms and those positive for LF/SOD-3) and with PI filter (see results), any out-of-focus spermatozoa noted in a frame were captured in subsequent frame with the appropriate focus. The captured frames were saved and later were meticulously examined to count the proportion of sperm and determine whether each sperm was stained for only LF/SOD-3, only PI, both or neither. To do this, each stage was captured with visible light overlaid with fluorescent light for DayLight488, the visible light was turned off and the filter was switched for PI and the same stage was captured without changing the focus. When some spermatozoa were out of focus, the same stage was re-focused, and the capture was repeated. The proportion of spermatozoa stained or not was then calculated in relation to the total number of sperm seen with visible light in the captured images. Spermatozoa negative for PI were considered live sperm as PI is an accepted method for determining the viability of many cell types including sperm cells. 

### 2.7. Statistics 

Data were analyzed by general ANOVA in the Statistics program (Analytical Software, Tallahassee, FL, USA). Stallions, ejaculates and treatments were included in the model as independent variables and significance was set at *p* < 0.05. For flow cytometry data, log transformation was used for analysis because the normal probability plot was skewed to the right, but the original means are reported in the figure and text. 

## 3. Results

**Figure 2:**
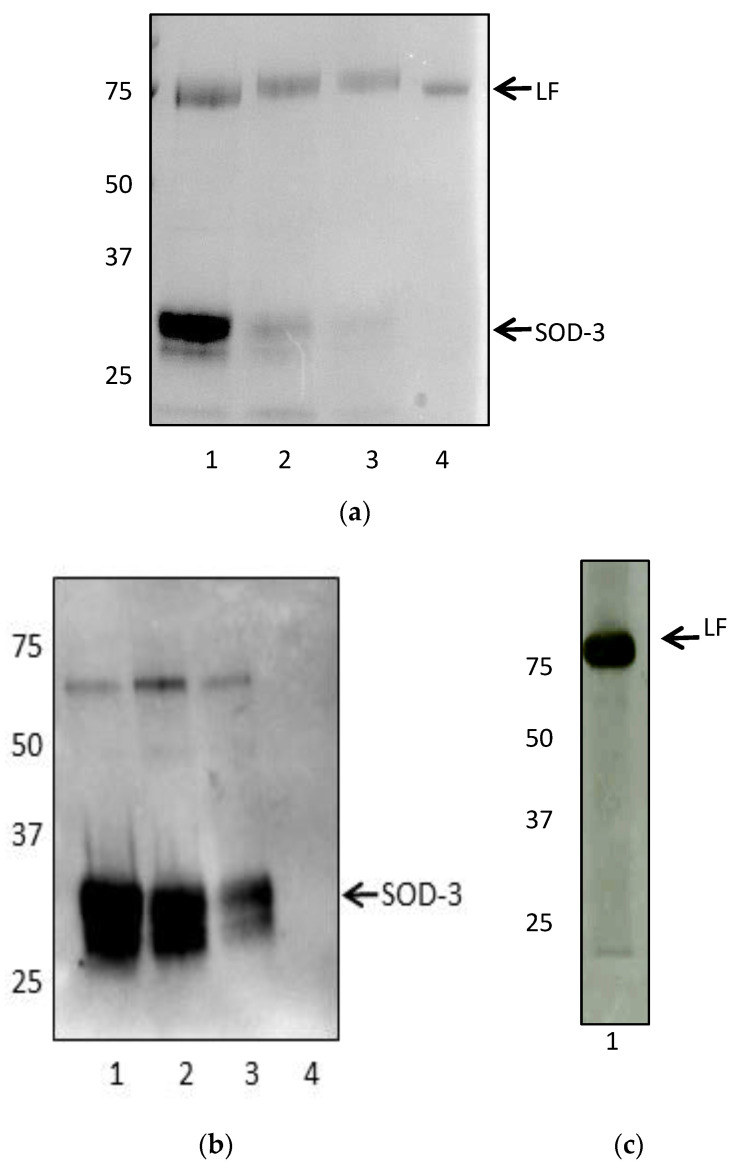
(**a**) SDS-PAGE of multiple attempts to isolate lactoferrin (80 kDa) from seminal plasma (lanes 1–3). A second protein at 32 kDa consistently co-eluted with lactoferrin. Lane 4: Recombinant human lactoferrin. (**b,c**): Western Blots of SOD-3 LF. (**b**) Western blot using anti-SOD-3 antibody (1:1000, SCBT, sc-67088) identified the second protein as SOD-3. Lanes 1–3: Lactoferrin purified using different methods. Lane 4: Recombinant human lactoferrin. (**c**) Western Blot using anti-lactoferrin antibody (1:1000, SCBT, sc-14434) of purified lactoferrin (lane 1).

Lactoferrin isolation was confirmed based on SDS-PAGE and Western blot, but in spite of additional approaches to separate lactoferrin from SOD-3 following the protocol of Inagaki et al. [32], SOD-3 was consistently present in the concentrated elution suggesting that the two proteins are tightly bound in equine seminal plasma (Figure 2).

**Figure 3:**
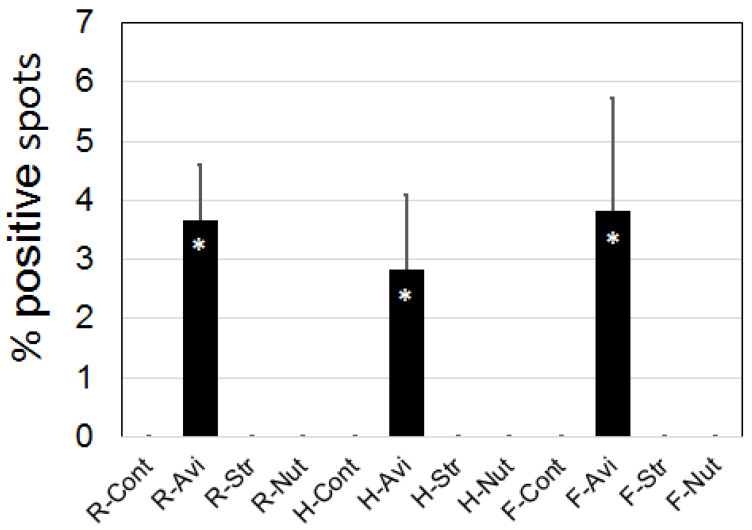
Avidin visible fluorescent spots. Positive spots were counted as a proportion of sperm seen with visible light. Asterisk indicates a significant difference *p* < 0.001. Abbreviations: R = room temperature; H = Heated; F = Snap-frozen; Cont = control; Avi = Avidin; Str = Streptavidin; Nut = NeutrAvidin.

Endogenous biotin was not detected on equine sperm under the conditions of this experiment, and this included heated or freeze-killed sperm with induced-membrane damage due to exposure to internal biotin. Positive staining for Avidin was initially suspected (averaging 3.44 spots in proportion to sperm number; Figure 3) and was significantly higher than that seen with the Streptavidin or NeutrAvidin incubation (*p* = 0.0001). However, these positive spots were found to be debris (false positives) and not associated with spermatozoa as confirmed with higher magnification. Streptavidin and NeutrAvidin showed no positive staining regardless of sperm conditions.

**Figure 4:**
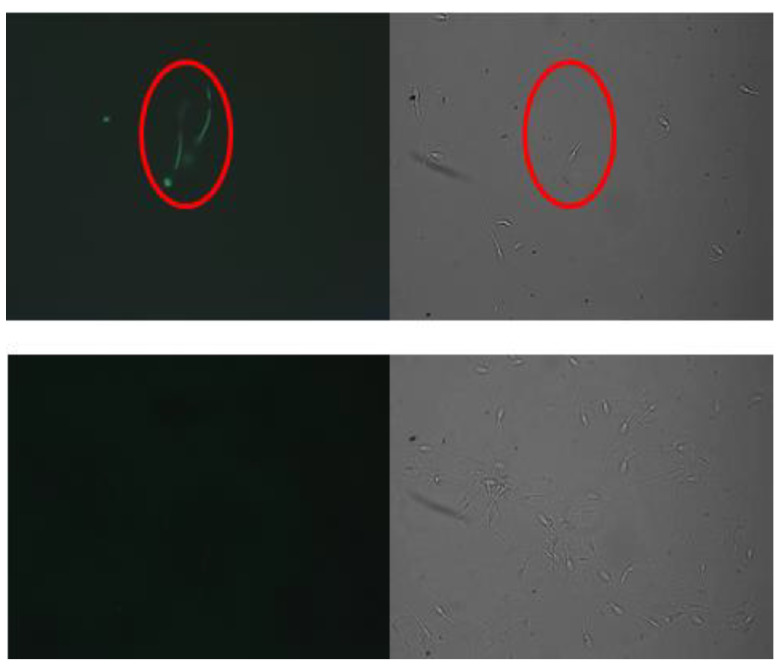
Fluorescent (**left panel**) and visible light (**right panel**) of the same microscopic field (200× magnification). The images show that blocking with BSA for as little as 10 min before the addition of biot-BSA did not inhibit biot-LF/SOD-3 binding (**upper panel**) but resulted in the inhibition of biot-BSA binding to sperm (**lower panel**) indicating the specificity of LF to its receptors. The red circle identifies the same spermatozoa.

Sperm blocked with 1% or more BSA for at least 10 min before the addition of biot-BSA were negative. Reducing BSA to less than 1% or for less than 10 min resulted in positive staining with biot-BSA that increased in intensity as the BSA concentration or blocking time was reduced. However, blocking non-specific sites with as much as 5% BSA or for as long as one hour did not inhibit or reduce positive staining with biot-LF/SOD-3 (Figure 4). Treatment of sperm with fluorescent NeutrAvidin without biotinylated protein resulted in negative staining. The addition of BSA at the same time as the biotinylated proteins (biot-BSA or biot-LF/SOD-3) resulted in similar staining intensity, indicating that non-specific binding occurs. To confirm the specificity, seminal plasma (which includes LF) was used to block biot-LF/SOD-3. This abrogated the positive staining, but seminal plasma was not able to block the nonspecific binding of biot-BSA in spermatozoa not pre-blocked with BSA. 

**Figure 5:**
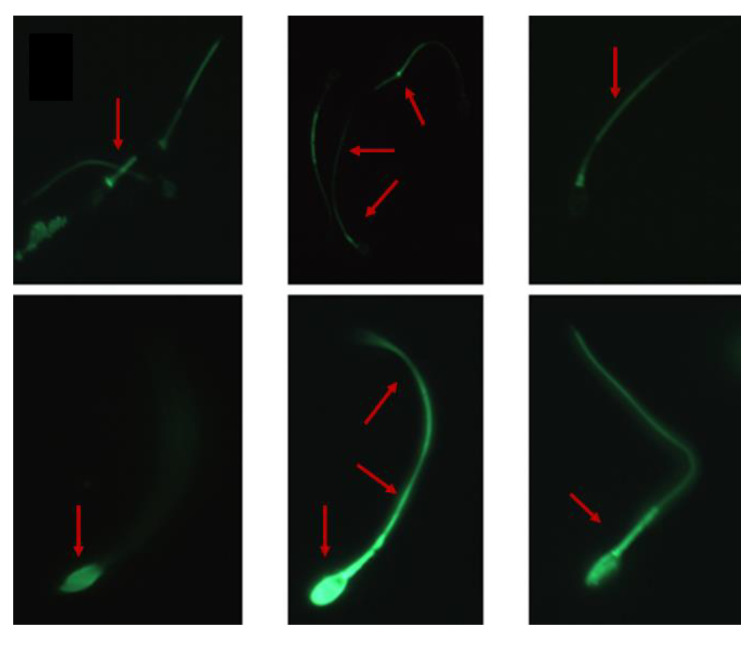
LF/Sod-3 binding to equine spermatozoa. Arrows show variations of LF/SOD-3 binding to different locations on equine sperm (600× magnification).

Generally, the binding sites for LF/SOD-3 on equine sperm included the head and tail with what appears to be a gap in the post-acrosome or the neck regions, but variations of this pattern were common where only part of the sperm was stained (tail or head) or where the staining was very intense all over the spermatozoa (Figure 5).

**Figure 6:**
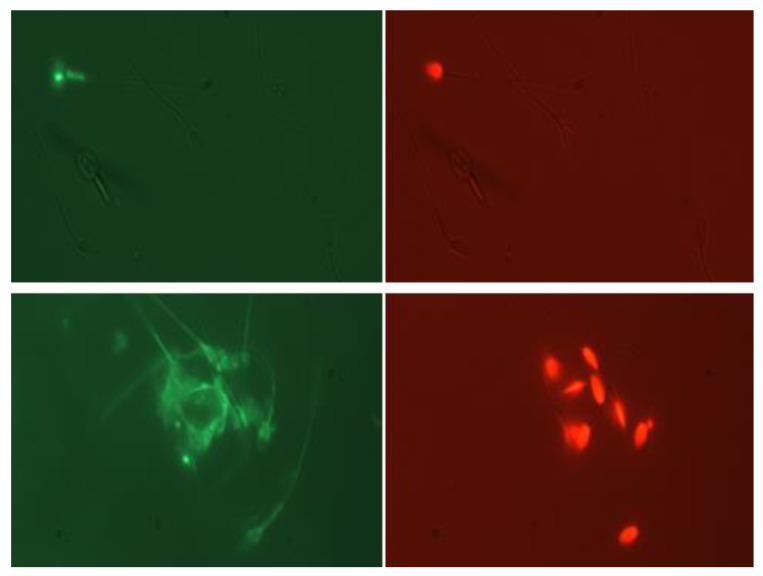
Visible light combined with a fluorescent microscopic image (400× magnification) of spermatozoa stained with DayLight488 (NeutrAvidin fluorescent conjugate; (**left panel**)) of the same sperm shown with PI staining (**right panel**). Non-treated (mostly viable sperm; (**upper panel**)) and snap-frozen (nonviable sperm; lower panel) showing PI+ sperm to be LF+; note that sperm that were PI− were predominately LF− (**upper panel**).

The microscopy-based sperm counting method for spermatozoa staining with PI and DayLight 488 in a proportion to the total number of sperm was found to be effective (Figure 6) and produced similar results to those seen with flow cytometry (Figure S1). 

**Figure 7:**
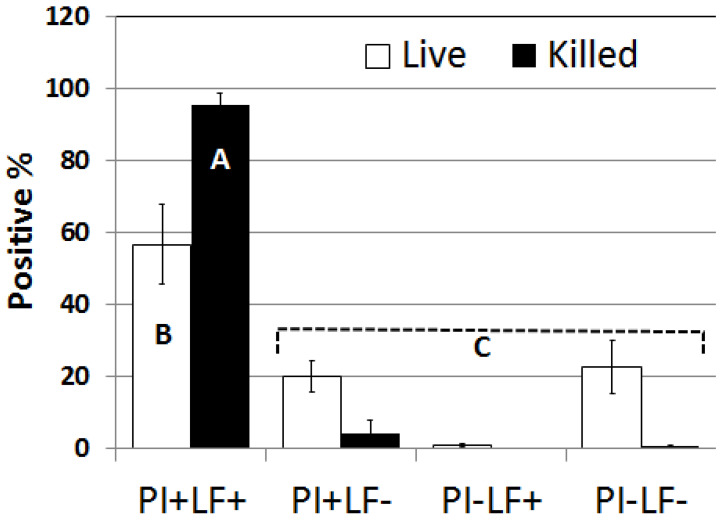
The proportion of untreated (live) or killed (snap-frozen) sperm positive for PI or LF counted from the image captured with the combined visible/fluorescent image seen in Figure 6. Note that almost all killed sperm were PI+ and LF+ (PI+LF+), but untreated sperm had a lower percentage of this category. On the other hand, untreated sperm had more than 20% PI negative that were also LF− (PI−LF−). Additionally, note that while untreated sperm had about 20% PI+ they were still LF− (PI+LF−), which may suggest that LF binding to sperm is sequential over time; i.e., as sperm deteriorate more, more LF will bind to them. Different letter (A.B.C) indicates significant differences *p* < 0.001.

**Figure 8:**
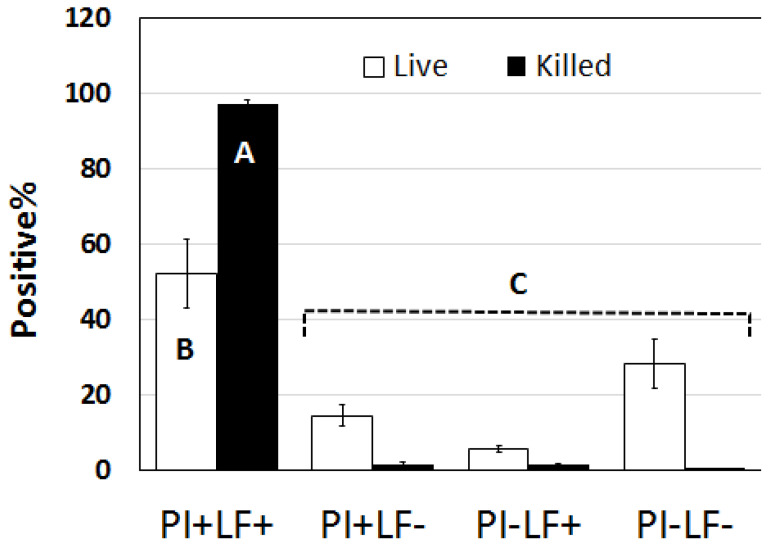
The proportion of untreated (live) or killed (snap-frozen) sperm positive for PI or LF counted by flow cytometry with very similar results to that seen with visual microscopic count (Figure 7) lending more credence to the validity of our observations. Different letter indicates significant difference *p* < 0.001.

The results for this method showed that spermatozoa classified as nonviable (subjected to snap freezing and thawing) were completely dead (99.6%; as indicated by their positive staining with PI; PI+) the majority of which were LF/SOD-3-positive (LF+; Figure 7). Only a small proportion (4.3%) of these PI+ sperm were LF/SOD-3-negative (LF−). In contrast, untreated spermatozoa had 76.8% positive staining with PI but only 56.7% were LF+. It should be noted that by the time spermatozoa were examined under the microscope, motility was always less than 10%, presumably as a result of multiple centrifugations. Motile sperm were almost exclusively negative (99.3%) for PI and LF/SOD-3 staining (only two out of more than 300 motile sperm had weak staining with LF). The flow cytometry results further confirmed microscopy, with the majority of killed sperm being PI+ and LF+ while the vast majority of untreated sperm that were PI− were also LF− (Figure 8).

## 4. Discussion

Sperm-neutrophil binding is an important facet of breeding and significantly impacts fertility. While a specific seminal plasma protein has been found to reduce this binding and improve fertility (CRISP-3), additional molecule(s) appear to promote binding between defective sperm and neutrophils. In this study, the binding of the LF/SOD-3 protein complex selectively to nonviable spermatozoa was determined by fluorescent microscopy as well as flow cytometry, and the results from the two methods were in agreement. Under the condition of this experiment, it was not possible to determine if the preferred binding of isolated and purified seminal plasma protein(s) to dead spermatozoa was due to lactoferrin, SOD-3, or a combination of the two proteins. While SOD-3 has been found in seminal plasma, it is believed to act as an antioxidant and no other function has been suggested for this protein. In contrast, LF has previously been associated with a variety of immune functions, including cytokine signaling, neutrophil chemotaxis, and antimicrobial activity (12,15). It is, therefore, likely that the observed effect on PMN-sperm binding was due to LF, but without separation of the two proteins and evaluation of each of the proteins, this cannot be concluded with certainty. To the best of our knowledge, this is the first report to show that equine seminal LF/SOD-3 binds to stallion sperm with specificity, due to the presence of LF and/or SOD-3 binding sites on equine sperm. The general pattern of LF/SOD-3 binding to equine sperm is similar to that reported for LF with human sperm [30], but the variation documented in this study may indicate serial exposure of its receptors/binding sites; that is, as sperm deteriorates further, more binding sites/receptors may be expressed on the sperm and eventually the whole sperm will be covered by the LF/SOD-3 complex. This may explain why a subset of sperm stained positive for propidium iodide (a marker for cell death) yet negative for LF, but to ascertain this observation, further experiments that specifically address this question are needed. 

The observation that seminal LF/SOD-3 binds to nonviable more than viable sperm supports the hypothesis that there is a physiological mechanism to discern between sperm populations [33]. We have previously described that seminal plasma protein CRISP-3 suppresses binding between PMNs and viable sperm in vitro, with no effect on non-viable sperm [4,7]. Furthermore, non-viable spermatozoa bound to PMNs at a significantly higher rate in the presence of seminal plasma [7]. Here, we report that motile sperm were almost exclusively negative for LF/SOD-3, the vast majority of PI^+^ sperm were positive for LF/SOD-3, and that the majority of PI^−^ sperm were negative for LF/SOD-3. Taken together, these observations suggest that LF/SOD-3 promotes PMN-binding and elimination of non-viable sperm from the mares’ reproductive tract, while CRISP-3 selectively protects viable spermatozoa from elimination. A selective suppression of PMN binding and phagocytosis of viable spermatozoa would allow for effective sperm elimination of dead and abnormal sperm from the uterus without interfering with the transport of sperm to the oviduct in the presence of ongoing inflammation. This would clearly be beneficial for effective and simultaneous sperm transport and elimination from the mare’s reproductive tract. 

The strong affinity and specificity of Biotin-avidin interaction are commonly exploited to facilitate the detection, purification, and labeling of different molecules [34]. Biotinylated protein can be used in a reaction and then probed with fluorescent avidin, offering an alternative detection assay when equine-specific antibodies are unavailable or difficult to make [2]. However, some cell types contain endogenous biotin, which can cause high background noise to confuse the sought signal [35]. To the best of our knowledge, there have been no previously published reports on whether mammalian sperm and specifically stallion sperm, contain endogenous biotin. Prior blocking of the non-specific binding sites on sperm by as little as 1% BSA completely abrogated the non-specific binding of biot-BSA to sperm but blocking these non-specific sites with as much as 5% BSA did not inhibit biot-LF binding. Furthermore, the specificity of LF binding was examined by blocking LF binding sites with fresh seminal plasma (which includes approximately 150 ug/mL LF) and incubating sperm with as little as 10% seminal plasma for as short as 10 min before the addition of biot-LF completely abolished biot-LF binding to sperm.

The results of this experiment suggest that biotinylated proteins should be considered when assessing equine sperm as long as appropriate controls are included. However, we recommend avoiding avidin when working with equine spermatozoa to prevent false positive results especially when microscope examination is not employed carefully, and instead to replace avidin with either Streptavidin or NeutrAvidin. Biotinylation of seminal plasma proteins offer an efficient method to study binding of sperm when compared to antibodies in terms of time and cost of production and/or optimization. While we cannot exclude the possibility that sperm mitochondria contain some endogenous biotinylated proteins [36], it did not appear to interfere with the biotinylated seminal plasma proteins used within this study, possibly indicating that the damage from heating and snap-freezing were not sufficient to expose these internal proteins. We also hypothesize that biotinylation of seminal plasma proteins (e.g., LF or CRISP-3) will provide us with a more efficient tool for identification and isolation of specific receptors from sperm membrane extracts; biotinylated seminal plasma proteins can be allowed to interact with extracted proteins from sperm membrane and capturing the complex with NeutrAvidin-conjugated column for downstream applications such as protein sequencing.

We conclude that binding of the seminal plasma LF or possibly, but less likely, SOD-3 appears to be selective towards nonviable sperm, potentially alluding to a physiological pathway for sperm selection and clearance. Furthermore, LF or SOD-3 receptors/binding sites are localized throughout the cell surface of equine spermatozoa, and this can be detected using biotinylated seminal plasma proteins. Finally, this label may occur in a sequential fashion as sperm membrane integrity and metabolism is continually stressed. 

## Data Availability

No public database is available for this study.

## Supplementary Materials

The following supporting information can be downloaded at: https://www.mdpi.com/article/10.3390/ani13010052/s1, Figure S1: Flow Cytometry of viable (not snap-frozen; upper panel (a) and nonviable sperm (snap-frozen; lower panel (b) for gate set up and quadrant-based background fluorescence elimination. Ab-breviations: Spz = sperm; PI = Propidium iodide; Avi = NeutrAvidin; LF = Lactoferrin. Plot 7 shows all sperm from the gate in plot 5; Plot 6 show sperm treated with NeutrAvidin but without LF. Plot 8 shows sperm treated with PI but without LF. Plot 9 show sperm treated with both PI and NeutrAvidin but without LF. Plot 10 show sperm treated with both PI and NeutrAvidin after treatment with biot-LF. All treatment were pre-blocked with 1% BSA before the addition of biot-Lf and all were subjected to the same centrifugation and washing even if LF was not used.
