# Supplementary material for: Binding of Equine Seminal Lactoferrin/Superoxide Dismutase (SOD-3) Complex Is Biased towards Dead Spermatozoa
Source: Animals (Basel). 2022 Dec 23;13(1):52. doi: 10.3390/ani13010052 (PMC9817809; doi:10.3390/ani13010052)
Supplement: Supplementary file 1 [file animals-13-00052-s001.zip › animals-2045571-supplementary.pdf]

Supplemental Figure S1

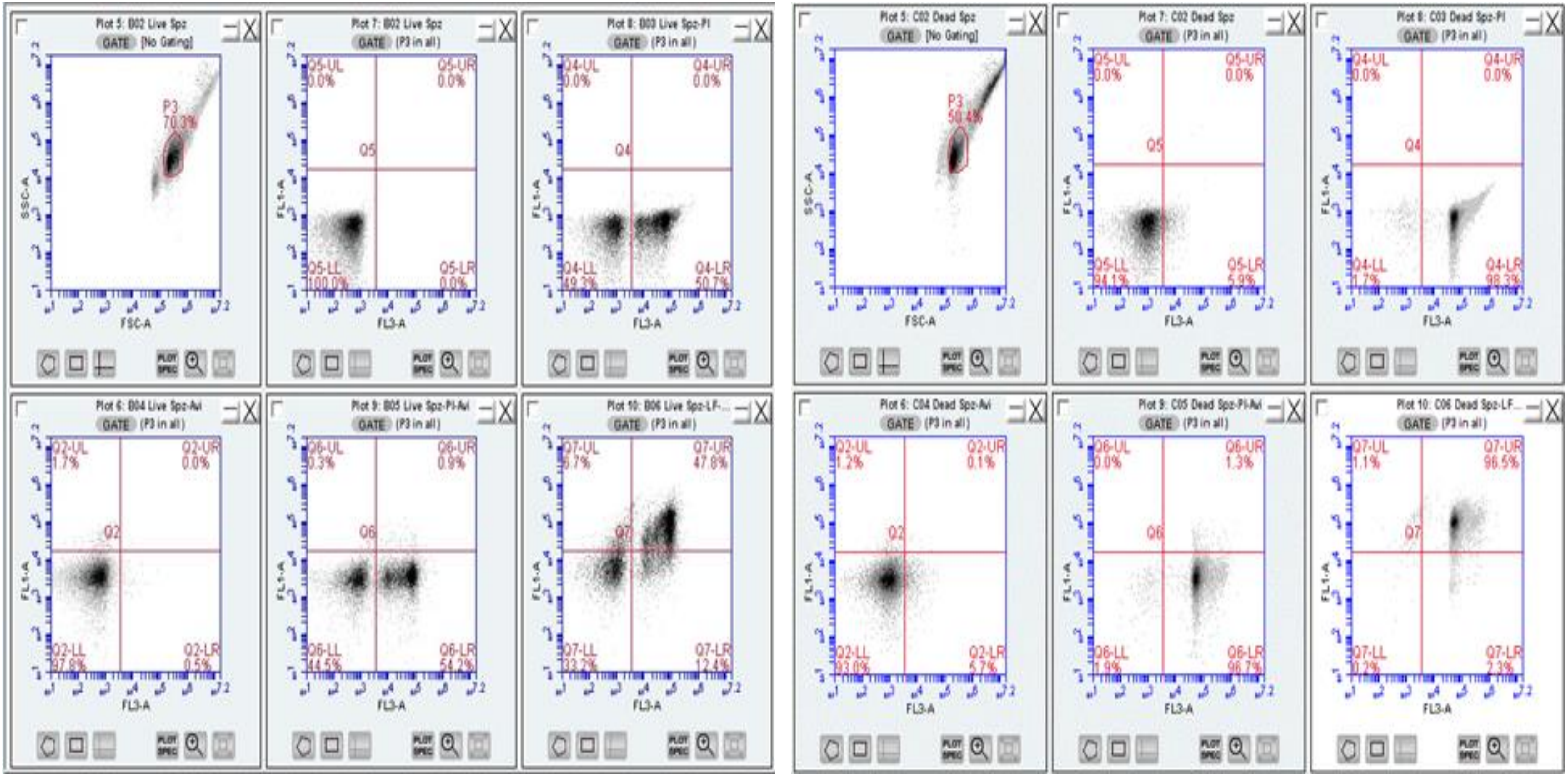

a

b

Supplemental Figure S1. Flow Cytometry of viable (not snap-frozen; left panel a) and nonviable sperm (snap-frozen; right panel b) for gate set up and quadrant-based background fluorescence elimination. Abbreviations: Spz = sperm; PI = Propidium iodide; Avi = NeutrAvidin; LF = Lactoferrin. Plot 7 shows all sperm from the gate in plot 5; Plot 6 show sperm treated with NeutrAvidin but without LF. Plot 8 shows sperm treated with PI but without LF. Plot 9 show sperm treated with both PI and NeutrAvidin but without LF. Plot 10 show sperm treated with both PI and NeutrAvidin after treatment with biot-LF. All treatment were pre-blocked with 1% BSA before the addition of biot-Lf and all were subjected to the same centrifugation and washing even if LF was not used.
